# The Ockham’s razor for estimating the needs of ICU beds during a pandemic

**DOI:** 10.1186/s13613-021-00874-w

**Published:** 2021-06-12

**Authors:** Pierre Squara

**Affiliations:** grid.477172.0 ICU, Clinique Ambroise Paré, 27bd Victor Hugo, 92200 Neuilly-sur-Seine, France

**Keywords:** COVID, Statistics, Resource

## Abstract

**Background:**

It is possible to monitor an epidemic evolution by plotting the number of patients, or its log-transform, as a function of time. However, these representations do not allow quick identifications of significant changes in the outbreak; a key information for estimating the needs for hospital and ICU beds, for decision-making, and resource allocation. Moreover, an epidemic is characterised by a heterogeneous evolution that depends on many unpredictable factors, coming from the virus itself or from its ecosystem. Simulations are very complex and based on hypotheses that are impossible to certify a priori, since each outbreak is different and has specific characteristics. A validation phase is necessary that may delay the usefulness of these tools. We tested a simpler method for monitoring the epidemic and rapidly predicting the peak.

**Results:**

We present here a simple and easy-to-draw figure by plotting the daily rate of change in the number of patients as a function of time. This allows: (1) to rapidly identify the changes in the infection growth, (2) to extrapolate the regression lines for predicting the peaks, and (3) to use simple statistical models for identifying the significant inflexions and deriving the uncertainties. This figure predicted confidently the peak epidemic of the three waves in France.

**Conclusion:**

Plotting the daily rate of change in the number of patients as a function of time is a simple tool for monitoring an epidemic growth, allowing to quickly identify significant changes and to help in predicting the peak of the infection, with its confidence interval.

The epidemic growth of a viral disease outbreak is most often characterised by the basic reproduction number R_o_, defined as the expected number of secondary cases following the introduction of one infectious individual into a fully susceptible population [1]. As a consequence, the number of infected patients (*N*) at a given moment in time (*t*) is modelised by an exponential curve *N* = *e*^ln(Ro).*t*^, where the unit of t is the time during which the patient is infectious. It can be seen from this formula that an outbreak must have *R*_o_ > 1 to invade a host population, otherwise it disappears right after its introduction.

While the infection is growing, the number of immunised patients also grows, leading to decrease the initial *R*_o_ down to an effective, time dependent, *R*_t_; therefore, changing the original curve into subexponential curves (series of exponential curves with time decreasing argument: *N* = *e*^ln(Rt).*t*^, with *R*_t_ < *R*_o_). Moreover, the population reacts to protect themselves against the infection (wearing masks, decreased social interactions, vaccinations, etc.…), leading to an additional decrease of the exponential argument. In contrast, the occurrence of clusters, more infective variants, or a decline in the host population protection may increase the infection growth once again, leading to a super exponential curve (series of exponential curves with time increasing argument up to the original *R*_o_ or to a new one).

It is possible to monitor the epidemic evolution by plotting *N* or log *N* as a function of time, as seen in Fig. 1. However, these representations do not allow a quick identification of the acceleration or deceleration of the outbreak, a key information when estimating the needs for hospital beds, especially in ICU, for decision-making and resources allocation. Moreover, an infection like the SARS Cov2 COVID-19 pandemic is characterised by a heterogeneous evolution that depends on many unpredictable factors coming from the virus itself or from its ecosystem. Simulations may become very complex and based on hypotheses that are impossible to certify a priori, since each outbreak is different and has numerous specific characteristics. A validation phase is necessary that may delay the usefulness of these tools [2, 3].

**Fig. 1:**
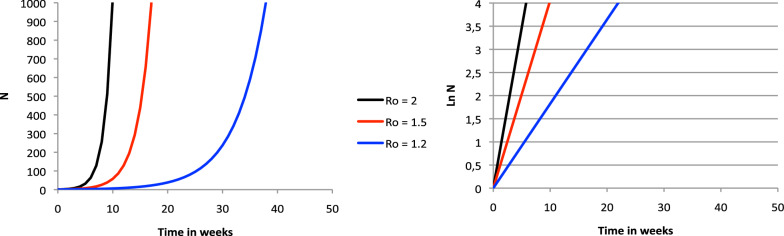
Theoretical evolution of the number of infected patients for three different *R*_o_, assuming that the infectious time is 1 week. Exponential curves may be linearised by plotting Log *N* (right panel) on the y-axis instead of *N* (left panel)

Therefore, applying the old principle of parsimony and simplicity (Ockham’s razor) may be of interest to obtain fast responses. We present here a simple and easy-to-draw figure allowing: (1) to quickly identify the inflexions in the infection growth, (2) to extrapolate the curves for predicting the peaks, and (3) to use simple statistical models for identifying the significant inflexions and deriving the uncertainties.

## A simple representation…

**Fig. 2:**
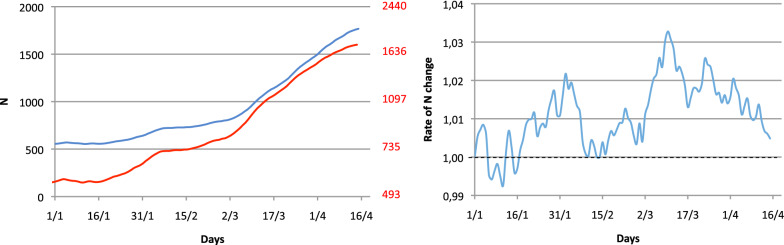
Evolution of the number of COVID-19 patients requiring intensive care (7 days moving average) in the Paris area. On the left panel, *N* is plotted in the y-axis with standard scale (blue curve) or log scale (red curve). On the right panel, the daily rate of *N* change is plotted on the y-axis)

The Fig. 2 shows the time evolution of the number (*N*) of COVID-19 patients who were hospitalised in all ICUs in the Paris area from January 1 to April 15. When plotting *N*, even with a log scale (left panel), it is not immediately clear if the epidemic is accelerating or decelerating.

We suggest illustrating the data by plotting the daily rate of change as a function of time. By doing this, an exponential curve becomes flat and the value on the y-axis is *R*_t_, if the time scale is appropriate. A super-exponential curve is thus linearly increasing and a sub-exponential curve is linearly decreasing (Fig. 2, right).

**Fig. 3:**
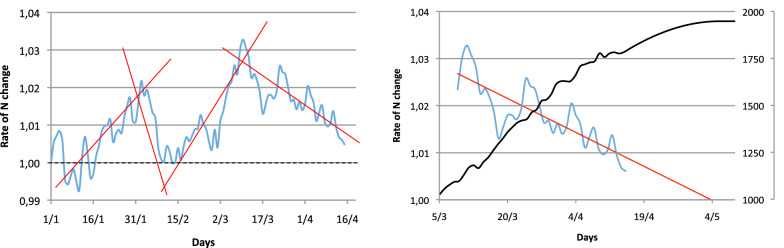
The left panel reports the same data as the right panel of Fig. 2, with identifications of significant slopes (red lines). When the curve reaches 1, the epidemic decreases (*R*_t_ < 1). On the right panel, we zoomed in on the last part of the evolution with its regression line and extrapolation crossing the x-axis on May 4. On this same figure, the corresponding number of patients can be derived from the regression curve and superimposed (black curve). The 95% confidence interval of both the regression line and the derived number of patients can be easily obtained from traditional regression lines models (not reported here for clarity)

This presentation also has the advantage of allowing the use of all statistical tools based on linear models. It is, therefore, easy to identify the presence of different regression lines. In the real example shown in the right panel of the Fig. 2, the least sum of residuals may identify four different slopes (Fig. 3, left) that correspond to four different equilibriums in the time evolution of the epidemic. It is also easy to extrapolate a regression slope. When the extrapolation crosses 1 on the y-axis, its value on the time axis (x-axis) indicates the date of the peak epidemic (Fig. 3, right).

Although not a simulation, this simple projection predicted the end of the three epidemic waves very confidently (exact peak day for the first wave, 3 days of difference for the second wave). For the third wave shown in Fig. 3, a peak was reached on 15 of February but was followed by a sudden reincrease of the slope. This shows that extrapolating a regression slope is only pertinent if the equilibrium between the infection and the ecosystem is stable. If not, the curve evolution moves out of the confidence interval of the regression slope, indicating that a new event is changing the equilibrium. In this example, the sudden resurgence was secondarily attributed to the emergence of the UK variant. One of these events may lie on political decisions against the pandemic. A change in the slope may, therefore, be used to evaluate their impact.

## …but of major interest

ICU rooms require more space than other hospital units, a specific technical environment, complex equipment, and highly qualified caregivers. Therefore, the number of available ICU beds is limited by essence. In France, whereas 5415 ICU beds were initially available, 7148 COVID-19 patients were admitted during the first wave, requiring the transformation of most acute care units and post-anaesthetic care units, and a massive mobilization of physicians, residents, nurses, and auxiliaries. This effort was balanced by a massive deprogramming of non-emergency procedures. These “ephemeral” ICUs have saved the French Health Care system but their quality, as compared to regular ICU, was not certified. Anticipating quickly the outbreak speed and the ICU bed needs is therefore of major importance to make the right decisions before being overwhelmed. On the other hand, it is also of major interest to anticipate the decrease of the outbreak, to reallocate the resources to standard care as soon as possible and to calibrate appropriately the lockdown of the population. Moreover, from an epidemiologic point of view, the number if ICU patients are less likely to be polluted by extra medical considerations or hidden factors than other hospital statistics.

## Conclusion

We believe that plotting the daily rate of change in the number of patients as a function of time is an interesting tool for monitoring an epidemic growth, allowing us to quickly identify significant changes and to help in predicting the peak of the infection with its confidence interval.

## Data Availability

Not applicable.
